# Anemia Status Changes Among Patients With Obesity Following Bariatric Surgery

**DOI:** 10.7759/cureus.60500

**Published:** 2024-05-17

**Authors:** Fahimeh Soheilipour, Delaram Eskandari, Jamileh Abolghasemi

**Affiliations:** 1 Minimally Invasive Surgery Research Center, Department of Pediatric Endocrinology, Iran University of Medical Sciences, Tehran, IRN; 2 Medicine, Iran University of Medical Sciences, Hazrat Rasoul Hospital, Tehran, IRN; 3 Department of Biostatistics, Iran University of Medical Sciences, Tehran, IRN

**Keywords:** roux-en-y gastric bypass, one-anastomosis gastric bypass, sleeve gastrectomy, obesity, bariatric surgery, anemia

## Abstract

Objective

This study aims to investigate trends in anemia severity among patients with pre-existing anemia who underwent bariatric surgery due to obesity. It also examines how different bariatric surgery techniques impact anemia outcomes.

Methods

This prospective study included 280 patients aged 18 to 65 with obesity who underwent bariatric surgery. The patients were categorized into three groups based on the type of surgery: sleeve gastrectomy, one-anastomosis gastric bypass, and Roux-en-Y gastric bypass. Anemia severity was evaluated over a 12-month follow-up period. Chi-square tests were used to assess the homogeneity of baseline factors among the groups, and McNemar tests along with generalized estimating equations were used to compare anemia outcomes.

Results

Before surgery, the rates of moderate anemia across the three surgical groups ranged from 18.2% to 22.4%, with no cases of severe anemia observed. There was no significant difference among the groups (p=0.949). During the 12-month follow-up, the odds ratio for reducing anemia severity in the sleeve gastrectomy and Roux-en-Y gastric bypass groups were 2.13 and 1.91, respectively, compared to the one-anastomosis gastric bypass group. Additionally, the odds ratio for reducing anemia severity in patients with hypothyroidism was 1.84 compared to those without hypothyroidism.

Conclusion

The choice of bariatric surgery technique significantly affects anemia outcomes, with sleeve gastrectomy showing a higher success rate in reducing anemia severity. The role of hypothyroidism in anemia management also appears to be significant.

## Introduction

Obesity is a significant health challenge in global societies and is recognized as a primary factor in elevating the risk of chronic diseases, such as type 2 diabetes, cardiovascular disease, stroke, joint diseases, and many other health conditions [1]. According to the World Health Organization, in 2022, 39% of adults (over 1.9 billion people) were overweight, and 13% (over 600 million people) were obese [2]. In the first national survey in Iran in 2004, 57.2% of overweight individuals were women, while the prevalence of obesity in women and men was reported as 25.2% and 11.1%, respectively [3]. A meta-analysis conducted in Iran in 2014 found that 21.7% of individuals over 18 years old and 6.1% of individuals under 18 were obese [4].

Bariatric surgery (BS) is considered a primary solution for weight loss in patients who have not achieved success with non-surgical methods. Bariatric procedures such as sleeve gastrectomy (SG), one-anastomosis gastric bypass (OAGB), and Roux-en-Y gastric bypass (RYGB) have been shown to lead to significant weight loss and improvement in obesity-related chronic diseases [5-8]. In SG, which is one of the minimally invasive BS methods, the stomach volume is significantly reduced. In RYGB, the stomach is divided into two sections, allowing food to bypass a portion of the digestive tract [9]. OAGB is similar to RYGB but creates a smaller stomach pouch and connects a segment of the small intestine to it, reducing nutrient absorption and potentially leading to malabsorption-related complications, such as iron and vitamin B12 deficiencies [10].

Nutritional deficiencies can cause a wide range of health problems. Anemia refers to a reduced concentration of hemoglobin, leading to decreased oxygen delivery to cells, which can impact physiological functions [11]. Individuals with obesity may develop anemia due to restricted intake of nutrient-rich foods, malabsorption disorders, and chronic inflammation [12]. Additionally, BS can lead to nutrient malabsorption, resulting in complications like anemia [13]. However, some studies indicate that BS can improve anemia in certain obese patients. A study by Bjørklund et al. showed that bariatric surgeries could reduce inflammation, lower serum hepcidin levels, and increase iron absorption, resulting in positive changes in anemia status [14].

This study aims to examine the changes in anemia severity among patients with obesity who have undergone BS. By tracking these changes over a 12-month period, we aim to understand how different types of BS impact anemia and provide insights into potential interventions for managing anemia-related complications.

## Materials and methods

Data collection

Data for this longitudinal study were collected from patients who visited the Obesity Clinic at the Minimally Invasive Surgery Research Center at Hazrat Rasoul Akram Hospital in Tehran between 2009 and 2023. These patients were unsuccessful with non-surgical weight loss methods and subsequently underwent one of three types of BS: SG, OAGB, or RYGB. Before surgical intervention, all patients were evaluated by a multidisciplinary team of bariatric surgeons, endocrinologists, nutritionists, psychologists, and psychiatrists. Different BS techniques were explained to the patients, who then selected the type of BS based on the benefits and risks of each approach.

Sample size

This study adopted a census-based approach. Among the patients who underwent BS at the center between 2003 and 2023, 280 were diagnosed with mild to moderate anemia before surgery. Their data were analyzed in this study. To ensure the adequacy of the sample size for analysis, we considered a confidence level (1-α) of 95% and a statistical power (1-β) of 80%. Previous studies estimated the proportion of anemia in obese patients before BS to be 0.12 [15], with a precision (d) of 0.1. The calculated sample size was 83, which is smaller than the 280 cases analyzed, indicating that the sample size was sufficient for this study.

$n=\frac{\left ( Z_{1-\alpha /2}+Z_{1-\beta } \right )^{2}pq}{d^{2}}=\frac{\left ( 1.96+.84 \right )^{2}\left ( 0.12\times 0.88 \right )}{0.1^{2}}=83 \left ( 1 \right )$

Measures

The response variable in our study was anemia status, calculated separately based on the World Health Organization (WHO) definition using hemoglobin values for women and men. Mild anemia was defined as hemoglobin levels of 110-119 g/l for women and 110-129 g/l for men. Moderate anemia was defined as 80-109 g/l, and severe anemia as less than 80 g/l for both men and women. Hemoglobin values of 130 g/l and above for men and 120 g/l and above for women were considered non-anemic [16]. Other variables measured include age, gender, type of BS (SG, OAGB, or RYGB), obesity-related diseases (such as type 2 diabetes, hypertension, dyslipidemia, hypothyroidism, and menstrual irregularities), weight (in kg), and BMI (in kg/m²), which were assessed before BS and at three, six, and 12 months after BS.

Measurement of obesity

Obesity was assessed using the body mass index (BMI), calculated by dividing weight in kilograms by the square of height in meters [2]. Obesity is defined as a BMI of 30 kg/m² or greater, indicating an abnormal ratio of fat to muscle tissue with excessive fat accumulation. Obesity class II, with a BMI between 35 and 39.9, and class III, with a BMI of 40 or higher, represent severe and morbid obesity, respectively [17]. Inclusion criteria encompassed individuals aged 18 to 65 with anemia and either class III obesity or class II obesity with at least one significant obesity-related condition, such as diabetes, hypertension, hypothyroidism, dyslipidemia, or menstrual irregularities.

Calculation of % of total weight loss

The percentage of total weight loss (%TWL) was calculated as follows [18]:

$\%TWL_{After Surgery}= 100\times \left ( Weight_{Base} -Weight_{After Surgery}\right )/Weight_{Base}$ (2)

$Weight_{Base}$ refers to the patient's weight before BS, and $Weight_{After Surgery}$ refers to the weight at the 3rd, 6th, and 12th months post-surgery.

Statistical analysis

Data analysis was performed using R software version 4.3.2 (R Foundation, Vienna, Austria). The Kolmogorov-Smirnov test (with a significance threshold of p>0.05) confirmed the normality of continuous variables. We used the Chi-squared test, Fisher's exact test, ANOVA, and Tukey's post-hoc test to analyze data. To examine trends and assess the effect of confounding variables, we used generalized estimating equations (GEE) with independent correlation structures and determined model fit using the Akaike Information Criterion (AIC) [19]. A significance level of 0.05 was set for all statistical tests.

Study inclusion and exclusion criteria

Participants included individuals aged 18 to 65 with class III obesity or class II obesity with an obesity-related disease who underwent one of the surgeries SG, OAGB, or RYGB for the first time and provided written consent. The exit criterion was patient dissatisfaction with continuing participation in the study.

All participants read a statement that explained the purpose of the study and provided written informed consent before participating in the study. They were reminded that participation in the study was voluntary, confidential, and that the results would remain anonymous. Ethics approval was obtained from the Ethics Committee of Iran University of Medical Sciences (no. IR.IUMS.REC.1396.31919). In conducting this research, appropriate ethical principles and methods were strictly adhered to during the collection of data from the samples. We ensured that all procedures involving human subjects complied with the ethical standards outlined. Prior consent was obtained from all participants, and anonymity/confidentiality was maintained throughout the study.

## Results

Out of patients aged 18 to 65 with obesity who underwent BS at the Minimally Invasive Surgery Center at Rasoul Akram Hospital in Tehran, the capital of Iran, from 2009 to 2023, 280 were diagnosed with anemia before surgery. Of these, 22 (7.9%) underwent SG, 158 (56.4%) underwent OAGB, and 100 (35.7%) underwent RYGB.

Table 1 provides the minimum, maximum, mean, and SE for age and %TWL for each follow-up period and across the three BS groups. Analysis of variance (ANOVA) with Tukey's post-hoc test was used for comparisons. The results showed that the mean age was homogeneous among the three BS groups. However, the mean percentages for total weight loss (%TWL) at three, six, and 12 months varied significantly across the three types of BS. Tukey's post hoc test revealed that the mean %TWL in the OAGB group differed significantly from the other two groups (p<0.05), with OAGB consistently showing a higher mean %TWL compared to SG and RYGB at all three time points. In summary, the OAGB group displayed the highest %TWL across all three time points. The observed differences in %TWL across the three BS types were statistically significant, particularly at the sixth and 12th months. Despite these differences in weight loss, there was no significant difference in mean age across the groups.

**Table 1:** The mean age and percentage of total weight loss (%TWL) by type of bariatric surgery (BS) at the third, sixth, and 12th months post-surgery BS - bariatric surgery, SG - sleeve gastrectomy, OAGB - one-anastomosis gastric bypass, RYGB - Roux-en-Y gastric bypass, Min - minimum, Max - maximum, SE - standard error, F - F-value for ANOVA, P - p-value for significance

Variables	BS type	Mean	SE	Min	Max	F	P
Age	SG	40.8	1.92	26	62	0.161	0.851
	OAGB	40.5	0.80	19	64		
	RYGB	39.9	0.84	22	57		
%TWL_3_	SG	18.0	1.26	8.9	32.2	5.234	0.006
	OAGB	20.5	0.32	11.6	30.2		
	RYGB	19.1	0.43	8.7	30.0		
%TWL_6_	SG	23.4	1.61	13.7	38.8	7.860	<0.001
	OAGB	28.2	0.42	14.1	40.6		
	RYGB	26.5	0.62	14.4	41.4		
%TWL_12_	SG	27.5	2.43	11.3	47.1	7.938	<0.001
	OAGB	36.0	0.71	0.0	58.0		
	RYGB	34.3	0.85	16.9	58.6		

Table 2 presents the frequency distribution of preoperative anemia, gender, blood pressure, diabetes, dyslipidemia, hypothyroidism, and menstrual abnormalities in women, categorized by the type of surgery. The preoperative analysis of anemia severity among patients undergoing different BS techniques revealed a consistent distribution across all three groups. Approximately 80% of these patients had mild anemia, while the remaining 20% had moderate anemia, with no cases of severe anemia. Chi-square tests confirmed that the distribution of anemia severity was homogeneous among the surgical groups before surgery, suggesting that the baseline levels of anemia did not vary significantly based on the type of BS. This observation indicates that the preoperative anemia severity among these patients was similar across the different surgery types. Additionally, Chi-square tests indicated that other preoperative factors, such as gender, hypertension, diabetes, dyslipidemia, hypothyroidism, and menstrual irregularities, were also uniformly distributed among the surgical groups. This uniformity in preoperative characteristics across the different BS groups demonstrates that these patients share similar baseline profiles before surgery. However, this does not imply that the outcomes regarding anemia severity after surgery will be consistent across the different surgery types. The subsequent sections of this discussion will delve into how anemia severity trends change over a 12-month follow-up period for each surgical group. Understanding these preoperative similarities provides a solid foundation for assessing how different surgical techniques might lead to varying outcomes in terms of anemia severity and other related factors.

**Table 2:** Frequency distribution of gender, anemia, and obesity-related diseases by type of bariatric surgery SG - sleeve gastrectomy, OAGB - one-anastomosis gastric bypass, RYGB - Roux-en-Y gastric bypass, X^2^ - Chi-square statistic, P - p-value for significance, ^t^ - Fisher exact test

Variables	SG	OAGB	RYGB	X ^2^	P
	No (%)	No (%)	No (%)		
Anemia					
Mild	18 (81.8)	122 (77.7)	76 (77.6)	0.147^t^	0.949
Moderate	4 (18.2)	35 (22.3)	22 (22.4)		
Gender					
Female	20 (90.9)	143 (90.5)	95 (95.0)	1.343^t^	0.246
Male	2 (9.1)	15 (9.5)	5 (5.0)		
Hypertension					
Yes	6 (27.3)	39 (24.7)	24 (24.0)	0.104	0.948
No	16 (72.7)	119 (75.3)	76 (76.0)		
Type 2 Diabetes					
Yes	4 (18.2)	51 (32.3)	23 (23.0)	3.736	0.160
No	18 (81.8)	107 (67.7)	77 (77.0)		
Dyslipidemia					
Yes	6 (27.3)	56 (35.4)	36 (36.0)	0.635	0.747
No	16 (72.7)	102 (64.6)	64 (64.0)		
Hypothyroidism					
Yes	2 (9.1)	39 (24.7)	19 (19.0)	3.117^t^	0.218
No	20 (90.9)	119 (75.3)	81 (81.0)		
Menstrual Abnormalities					
Yes	3 (15.0)	30 (21.0)	21 (22.1)	0.387^t^	0.842
No	17 (85.0)	113 (79.0)	74 (77.9)		

**Figure 1:**
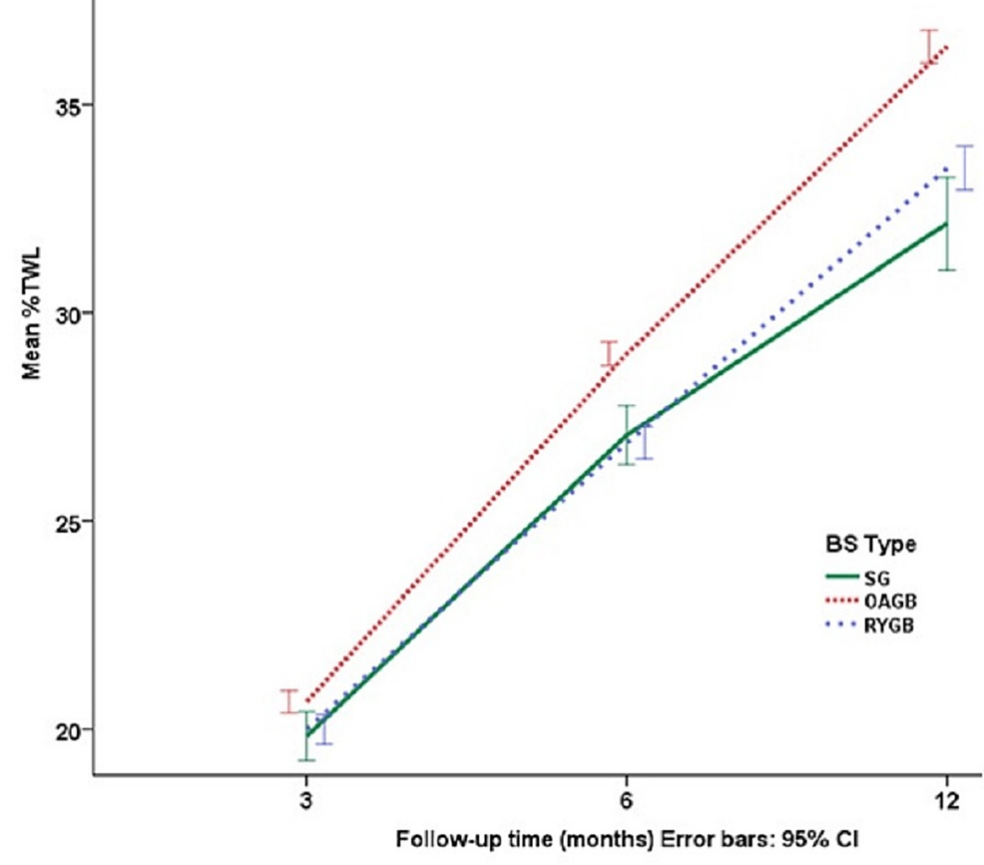
Mean %TWL by type of BS and follow-up times. This chart shows the mean percentage of total weight loss (%TWL) at three, six, and 12 months post-bariatric surgery, categorized by the type of BS SG - sleeve gastrectomy, OAGB - one-anastomosis gastric bypass, RYGB - Roux-en-Y gastric bypass

Figure 1 depicts the trend in the percentage of %TWL over the 12-month follow-up period. The trend is nearly linear, showing consistent weight loss over time. Notably, the highest percentage of total weight loss was observed in the OAGB group, suggesting that this surgical technique may be more effective in achieving significant weight loss compared to other BS types.

**Figure 2:**
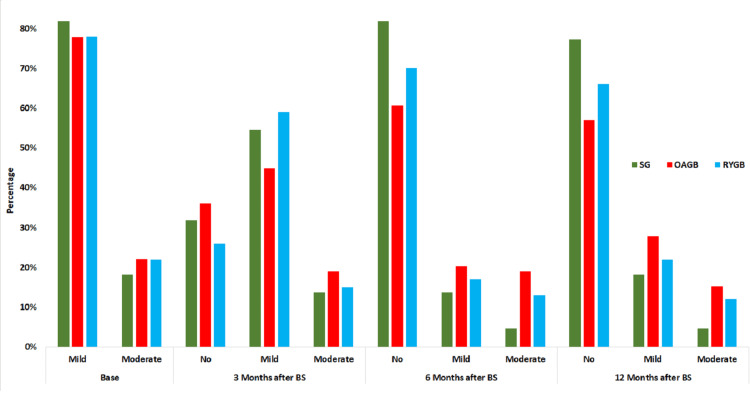
Trend in anemia severity by type of bariatric surgery and follow-up time SG - sleeve gastrectomy, OAGB - one-anastomosis gastric bypass, RYGB - Roux-en-Y gastric bypass

Figure 2 shows the trend of changes in the severity of anemia across the three surgical groups. At the preoperative baseline (base), the frequency distribution of anemia was relatively homogeneous across the three groups. After three months, a decrease in moderate anemia severity was observed in all three groups, with a more pronounced reduction in the SG and RYGB groups. After six months, the SG group exhibited a significant decrease in the moderate anemia severity, and this trend continued at the 12-month postoperative follow-up. Overall, the OAGB group had a lower percentage of samples with resolved anemia compared to the other two groups.

The comparison of changes in anemia severity relative to pre-BS anemia status, categorized by follow-up time and surgical group, is shown in Table 3. The McNemar test was significant in all groups and follow-up times. In the SG group, during the first three months after BS, one-third of patients with mild anemia and one-quarter of patients with moderate anemia showed improvement. By the second three months, approximately 95% of patients with mild anemia had improved. The slope of the decrease in anemia severity slowed down and stabilized by the 12-month follow-up. The OAGB group showed a similar trend in anemia severity changes, with one-third of patients with mild anemia and about half of patients with moderate anemia improving during the first three months. However, the slope of the decrease was less pronounced compared to the SG group. The RYGB group exhibited a similar pattern of changes, with an initial decrease followed by stabilization.

**Table 3:** Frequency distribution of anemia after BS by follow-up time and comparison with baseline BS - bariatric surgery, SG - sleeve gastrectomy, OAGB - one-anastomosis gastric bypass, RYGB - Roux-en-Y gastric bypass, P - p-value for significance

BS type	Time (months)	Anemia severity	Base time		McNemar-Bowker statistic	P
			Mild No (%)	Moderate No (%)		
SG	3 M	No	6 (33.3)	1 (25.0)	7.0	.030*
		Mild	12 (66.7)	0 (0.0)		
		Moderate	0 (0.0)	3 (75.0)		
	6 M	No	17 (94.4)	1 (25.0)	9.0	<0.001*
		Mild	1 (5.6)	2 (50.0)		
		Moderate	0 (0.0)	1 (25.0)		
	12 M	No	16 (88.9)	1 (25.0)	19.1	<0.001*
		Mild	2 (11.1)	2 (50.0)		
		Moderate	0 (0.0)	1 (25.0)		
OAGB	3 M	No	41 (33.3)	16 (45.7)	61.8	<0.001*
		Mild	64 (52.0)	7 (20.0)		
		Moderate	18 (14.6)	12 (34.3)		
	6 M	No	80 (65.0)	16 (45.7)	99.4	<0.001*
		Mild	20 (16.3)	12 (34.3)		
		Moderate	23 (18.7)	7 (20.0)		
	12 M	No	76 (61.8)	14 (40.0)	90.3	<0.001*
		Mild	30 (24.4)	14 (40.0)		
		Moderate	17 (13.8)	7 (20.0)		
RYGB	3 M	No	22 (28.2)	4 (18.2)	26.8	<0.001*
		Mild	52 (66.7)	7 (31.8)		
		Moderate	4 (5.1)	11 (50.0)		
	6 M	No	62 (79.5)	8 (36.4)	70.1	<0.001*
		Mild	7 (9.0)	10 (45.5)		
		Moderate	9 (11.5)	4 (18.2)		
	12 M	No	59 (75.6)	7 (31.8)	66.5	<0.001*
		Mild	11 (14.1)	11 (50.0)		
		Moderate	8 (10.3)	4 (18.2)		

Table 4 presents the results from the generalized estimating equations (GEE) analysis, which was used to determine the trend in anemia severity among the study patients. The final fitted model showed that, among the variables analyzed, only the type of surgery and hypothyroidism were significant at the 0.05 level. The Intercept values from the GEE model indicate that, under the same conditions of surgery type and hypothyroidism status, the odds ratio for transitioning from moderate anemia to no anemia is 4.18. Similarly, the odds ratio for changing from moderate anemia to mild anemia is 33.85. The GEE results also indicated that the odds of reducing anemia severity in the SG and RYGB techniques are 2.1 and 1.91 times greater, respectively, compared to the OAGB surgery. In other words, the SG and RYGB techniques are more effective in improving anemia status in patients following BS. Moreover, the odds ratio for reducing anemia severity after BS in the group with hypothyroidism is 1.84 times greater than in the group without hypothyroidism.

**Table 4:** Factors associated with the change in anemia severity using the GEE method SG - sleeve gastrectomy, OAGB - one-anastomosis gastric bypass, RYGB - Roux-en-Y gastric bypass, and %TWL - percentage of total weight lost, \beta - coefficient of variable, SE - standard error, $Wald=\left ( \beta /SE \right )^{2}$ , OR - odds ratio

Variables		β	SE	Wald	P	OR
Intercept	No	1.43	0.689	4.31	0.038	4.18
	Mild	3.52	0.700	25.33	0.001	33.85
	Moderate	0				1
Age		0.02	0.019	0.79	0.372	1.02
%TWL 3 M		-0.03	0.050	0.424	0.515	0.97
%TWL 6 M		0.02	0.051	0.143	0.705	1.02
%TWL 12 M		-0.02	.0268	0.358	0.550	0.98
Gender	Female	0.77	0.298	6.689	0.010	2.16
	Male	0				
Surgery type	SG	0.76	0.283	7.123	0.008	2.13
	RYGB	0.64	0.289	4.956	0.026	1.91
	OAGB	0				
Diabetes	Yes	0				1
	No	-.025	.2954	0.007	0.932	0.97
Dyslipidemia	Yes	0				1
	No	0.38	0.328	1.353	0.245	1.46
Hypertension	Yes	0				1
	No	0.14	0.298	0.223	0.637	1.15
Hypothyroidism	Yes	0				1
	No	.610	0.292	4.360	0.037*	1.84

## Discussion

This prospective study investigated changes in anemia severity in patients who underwent BS due to obesity. Among the three BS techniques studied, OAGB was most effective for weight loss, with 9% greater weight loss compared to SG and 2% greater than RYGB at 12 months post-surgery. Weight loss trends across the three BS types were generally linear, with OAGB demonstrating the steepest positive slope. These results are consistent with earlier studies showing the effectiveness of OAGB over other BS types [20,21].

In our study, the SG group experienced the most significant reduction in anemia severity, with nearly 95% of patients with mild anemia showing improvement after BS. The OAGB group had the highest %TWL but showed less consistent improvement in anemia severity, while the RYGB group showed initial improvement followed by a plateau. These findings suggest that while OAGB might be more effective for weight loss, SG appears to be more effective at reducing anemia severity. This could be due to the unique surgical approaches, with SG possibly causing fewer malabsorption-related issues. Further research is required to explore the mechanisms behind these results and validate them in larger cohorts [22].

In all three surgical groups analyzed, about 80% of our study patients had mild anemia, while the rest had moderate anemia. We did not encounter any cases of severe anemia. Chi-square tests showed that the distribution of anemia severity, gender, blood pressure, diabetes, dyslipidemia, hypothyroidism, and menstrual irregularities was homogeneous across the three surgical groups. Enani and colleagues conducted a systematic review to compare anemia rates in sleeve gastrectomy (SG) and Roux-en-Y gastric bypass (RYGB) techniques. They reported that pre-bariatric surgery anemia rates varied from 3.4% to 22.0% across 20 longitudinal studies with follow-up periods ranging from 12 to 120 months. In the SG group, anemia rates before and after surgery were 11.2% and 22.9%, respectively, while for the RYGB group, these rates were 19.1% and 19.6%. This suggests a relative advantage of the SG technique in terms of managing anemia among bariatric surgery patients [23]. These results align with our study's findings. To determine and compare anemia severity by type of BS and follow-up period, we used the McNemar test and Figure 2. The SG group showed a greater reduction in anemia severity compared to the other two groups. After BS, nearly 95% of the patients with mild anemia in the SG group experienced improvement. Despite this, SG showed a weaker performance in terms of percentage of %TWL compared to the other groups, indicating that weight loss in this group was more gradual. This milder weight loss could be a contributing factor to SG's success in improving anemia among patients.

Moreover, the coexistence of anemia and hypothyroidism highlights a critical clinical area. The connection between these two conditions is still unclear, but thyroid hormones influence erythrocyte production and erythropoietin levels, suggesting potential causal links. Our results showed that hypothyroidism might contribute to greater anemia severity after BS. Improvements in thyroid function could lead to reduced severity of anemia [24]. Future studies should investigate this relationship and its implications for managing anemia in BS patients. Additionally, the elevated levels of thyroid-stimulating hormone (TSH) observed in obese individuals tend to normalize with weight loss, indicating that weight loss can lead to improved thyroid function and potentially reduced anemia severity [25-27]. Therefore, comprehensive approaches that include monitoring thyroid function and ensuring appropriate supplementation of essential nutrients like iron, vitamin B12, and folic acid are crucial for managing post-BS anemia [28].

Limitations

Our longitudinal study design posed potential challenges related to data loss or participant attrition over time. To mitigate these limitations, several strategies were implemented. Firstly, we enhanced our data collection procedures by providing comprehensive training to participants, emphasizing the importance of consistent attendance at follow-up appointments and accurate reporting of any adverse events or unexpected reactions. Additionally, we actively engaged with participants via telephone calls and text messages to encourage their continued participation in the study and to minimize the risk of missing data. These efforts aimed to maximize retention rates and ensure the completeness of our dataset. Furthermore, we employed advanced statistical methods, such as generalized estimating equations (GEE) with ordinal logistic link, to address data correlation and minimize biases arising from repeated measurements. These measures ensured the robustness and reliability of our study findings despite the inherent limitations of longitudinal research designs.

## Conclusions

In conclusion, our study emphasizes the considerable impact of BS on anemia severity and its relationship with hypothyroidism. Our results indicate that SG is the most effective technique for reducing anemia severity, outperforming RYGB. While OAGB showed greater weight loss, its impact on anemia severity was not significantly different from RYGB. This suggests that SG may be a better choice for patients concerned about anemia-related outcomes after BS. Additionally, the co-occurrence of anemia and hypothyroidism underscores the importance of a comprehensive diagnostic approach and targeted treatment for these conditions. Given the multifaceted effects of thyroid disorders on anemia, further research is needed to understand the underlying mechanisms and validate our findings in larger studies. This research could help improve clinical guidelines for managing anemia in patients undergoing BS and those with hypothyroidism.
